# Effects of regulator of G protein signaling 2 (RGS2) overexpression in the paraventricular nucleus on blood pressure in rats with angiotensin II-induced hypertension

**DOI:** 10.3389/fphys.2024.1401768

**Published:** 2024-06-21

**Authors:** Shane H. Boomer, Xuefei Liu, Hong Zheng

**Affiliations:** Basic Biomedical Sciences, Sanford School of Medicine, University of South Dakota, Vermillion, SD, United States

**Keywords:** regulator of G-protein signaling 2, G-protein-coupled receptor, angiotensin-II, paraventricular nucleus, blood pressure, hypertension

## Abstract

The hypothalamic paraventricular nucleus (PVN) regulates sympathetic activity and blood pressure. The regulator of G protein signaling 2 (RGS2) is a negative G protein regulator, which selectively regulates G⍺q signaling, a potential cause of hypertension. This study aimed to examine angiotensin II (ANG II)-G protein-RGS2 signaling on the central mechanisms of blood pressure control, sympathetic activation, and kidney function. The Sprague Dawley rats were infused with ANG II (200 ng/kg/min) via osmotic mini pump to induce hypertension. Adenovirus (AV) vectors encoding RGS2 was transfected into the PVN *in vivo*. By radio telemetry measurements, we found AV-RGS2 transfection to the PVN significantly attenuated the increase of mean arterial pressure in ANG II infusion rats from days 2–7 of the 2-week experiment (Day 7: ANG II + AV-RGS2 141.3 ± 10.0 mmHg vs. ANG II 166.9 ± 9.3 mmHg, *p < 0.05).* AV-RGS2 transfection significantly reduced the serum norepinephrine level and acute volume reflex and increased daily urine volume and sodium excretion in ANG II-infused hypertensive rats. AV-RGS2 transfection significantly reduced G⍺q and PKC protein expressions within the PVN in ANG II infusion rats. In cultured mouse hypothalamic cells, real-time PCR study showed ANG II treatment increased mRNA expression of G⍺q, G⍺s, and RGS2, and AV-RGS2 treatment decreased ANG II-induced mRNA expression of G⍺q and G⍺s. Using confocal imagery, we found that AV-RGS2 attenuated the increase of calcium influx in ANG II-treated cells. Our results suggest that central overexpression of RGS2 in the PVN attenuated the increase of blood pressure and sympathetic outflow, and improves kidney excretory function in hypertensive rats. This may be via the alteration of ANG II-G-protein-RGS2 signaling in the central nervous system.

## Introduction

Hypertension is a cardiovascular disease that significantly increases associated mortality and morbidity (Chobanian et al., 2003; Wu et al., 2015). Data suggests that more than 50% of clinical hypertension cases are primary (essential) forms, often featuring a neurogenic origin (Frame et al., 2016). The hypothalamic paraventricular nucleus (PVN) is a critical central nucleus regulating sympathetic activity. Excitatory input to pre-autonomic neurons in the PVN leads to increased sympathetic outflow in animal models with hypertension (Li and Pan, 2017; Guyenet et al., 2020). The dysfunction of sympathetic modulation within the PVN contributes to fluid and electrolyte balance alterations, which are seen in animals with hypertension and chronic heart failure (Zheng et al., 2022). With its roles in stress, metabolism, growth, reproduction, and autonomic function, the PVN has been established as one of the most critical control autonomic centers in the brain (Ferguson et al., 2008; Zhang and Anderson, 2014).

The sympathetic nervous system (SNS) acts on ⍺- and β-adrenergic receptors that are coupled to G-proteins. Specific ⍺- and β-adrenergic receptors act on specific G-protein isoforms. ⍺1 receptors are coupled to Gq proteins, ⍺2 receptors are coupled to Gi proteins, and β1 and β2 receptors are coupled to Gs proteins (Vasudevan et al., 2011; Taylor and Cassagnol, 2024). Heterotrimeric G-proteins have three distinct subunits (⍺, β, and γ). As GTP binds, the ⍺ subunit dissociates from the β and γ subunits, triggering downstream signaling specific to the G-protein isoform. In the isoforms associated with excitatory function, the signaling cascade is an essential factor in increasing cellular activity, causing several proteins to be transcribed relating to cell cycle progression, proliferation, growth factors, metabolism, neurotrophy, and cell survival (Wang et al., 2018; Alshak and Das, 2024). With G-proteins being present within all cell types, it is plain to see their vital role in cell signaling. Understanding the regulatory mechanisms of different isoforms of G-proteins within the brain, specifically Gαq/Gαs, is essential in how they are directed to modify sympathetic activation and cardiovascular parameters.

Angiotensin II (ANG II) acts to activate type 1 (AT_1_) and type 2 (AT_2_) receptors, each with their corresponding mechanism. As ANG II binds to the AT_1_ receptor, a conformation change ensues, allowing GTP binding, subunit dissociation, and intracellular signaling (Eguchi et al., 2018). AT_1_ receptors are coupled to a Gq protein, where the ⍺ subunit will act to activate phospholipase C enzyme (Freeman and Tallant, 1994). This enzyme then acts to increase intracellular calcium through inositol triphosphate (IP3) and activate protein kinase C (PKC) through diacylglycerol (Bill and Vines, 2020). ANG II stimulates sympathetic activity, increases vasoconstriction, impacts sodium and water reabsorption in the kidneys, and influences baroreceptor sensitivity, stress responses, and cardiovascular remodeling (Fyhrquist et al., 1995).

The regulators of G-protein signaling (RGS) family regulates G protein signaling through their GTPase-activating proteins (GAP) activity. This GAP activity causes GTP to hydrolyze to GDP, thus inactivating and causing the reassociation of the three subunits (⍺, β, and γ), and halting the signaling cascade (Kimple et al., 2011). Further looking into RGS’s activity on G-proteins and the cessation of cell signaling may be an important feature in the progression of blood pressure dysregulation. Of the RGS protein family, RGS2 regulation has been shown to be an emerging target in multiple tissues for various pathologies (McNabb et al., 2020; O'Brien et al., 2019). Studies have examined RGS2 in cognitive dysfunction, cardiac fibrosis, and pancreatic dysfunction. With little to no studies being done on the RGS2 protein on G-protein signaling on the central mechanisms of blood pressure control, we view it as important to examine how these interactions affect sympathetic reflex and the progression of hypertension. Examining the neuronal activity with cell signaling cascade changes in blood pressure management between hypertensive and normotensive subjects may be a critical link needed to help better combat this condition.

The present study aimed to test the central molecular mechanisms of RGS2 protein and its relationships with the sympathetic nervous system, as well as its roles in cardiovascular and renal function. *In vivo*, we assessed the blood pressure changes, sympathetic activation, acute volume reflex, and kidney function in chronic ANG II infusion rats with central adenovirus (AV) RGS2 transfection in the PVN. *In vitro*, we assessed the changes in G-protein with ANG II and AV-RGS2 treatment. We also examined ANG II-induced calcium flux changes as we altered the expression of RGS2 in the neuronal culture cells.

## Materials and methods

### Animals

For *in vivo* study, we used male Sprague-Dawley rats (treatment started at 12 weeks of age, end-of-treatment aged approximately 14–16 weeks, total animal number = 60) of *Rattus norvegicus* (Envigo and USD Animal Resource Center). The rats were housed in the Animal Resource Center at the University of South Dakota on a 12-h day/night cycle with *ad libitum* access to standard rat chow and water. All the procedures on animals were approved by the Institutional Animal Care and Use Committee of the University of South Dakota. The experiments were in accordance with the American Physiological Society and the National Institutes of Health Guide for the Care and Use of Laboratory Animals.

### Anesthesia

For rat survival surgeries, the subject was placed unconscious via isoflurane gas introduced at 4%–5% in oxygen. Maintenance levels (2%–4%) of isoflurane were given, and reflexes were monitored to ensure adequate anesthetic dosage. Ventilatory rate, depth, and quality were assessed and titrated to ensure the proper anesthetic without loss of ventilation. Post-surgery, 0.1 mL buprenorphine SR (0.5 mg/mL) was given subcutaneously for analgesia. The subject was monitored following surgery for proper and timely surgical site healing.

In rat non-survival surgeries, the subject was given either urethane and ⍺-chloralose (0.5 mL/100 g, concentration 8.8 g/50 mL), or Thiobutabarbital (Inactin Hydrate) (2 mL/kg, concentration 0.05 g/mL) via intraperitoneal injection. Reflexes were monitored, and sacrifice occurred when reflexes were absent. Additional doses of anesthetic were given accordingly. The subject was sacrificed following the completion of the experiment.

### Chronic ANG II infusion via osmotic mini pump

In this survival surgery, the osmotic mini pump (Alzet: Model 2002; Durect, CA) was inserted into the scruff of the neck of the subject. An incision was made, and blunt dissection was performed to create a cavity where the osmotic pump was placed. The osmotic pump was calibrated to deliver ANG II (Sigma, St Louis, MO) at 200 ng/kg/min over 2 weeks.

### Adenoviral RGS2 (AV-RGS2) transfection in the PVN

The subject was anesthetized and placed on the stereotaxis device. An incision was made along the midline of the cranium. A small hole was drilled into the skull to allow for the insertion of the needle and delivering the injection into the PVN. To give an injection into the PVN, we moved from bregma −1.7 to −1.8 mm along the A-P axis, +/− 0.5 mm along the M-L axis and went to a depth of 7.5–8 mm from the surface of the skull. 50 nL of AV-RGS2 with co-expression of reporter mCherry (2 × 10e9, SignaGen, Frederick, MD) or AV-GFP was given bilaterally upon each injection. AV-RGS2 transfection, in relation to ANG II infusion, occurred on the same day as the osmotic minipump implantation.

Transfection evaluation was completed by RGS2 mRNA level, histological assessment confirming mCherry staining and RGS2 fluorescent signal within the PVN area. For RGS2 mRNA studies, additional rats were transfected with AV-GFP as a control. For histological studies of AV-RGS2 transfection, each subject was used as self-control. The AV-RGS2 was introduced unilaterally to the PVN region in the left hemisphere, with no virus being introduced to the right hemisphere. We compared the left *versus* right PVN by the presence and absence of AV-RGS2 coupled with mCherry fluorescence.

### Radio telemetry recording of blood pressure

The telemetry unit (Model PA-C10, Data Sciences International, St. Paul, MN) was inserted into the subject by making a subdermal pocket adjacent to the femoral artery. A magnet and radio were used to activate and assess the telemetry device. With telemetry implanted, we monitored blood pressure and heart rate changes before and during ANG II infusion in conscious states for 2 weeks. Blood pressure and heart rate measurements were recorded every hour in the morning to afternoon for 1 min and averaged for each day following the conclusion of the experiment. Data was recorded and stored on the Ponemah (Data Sciences International) outfitted for the mentioned telemetry devices. Measurements were taken over 2 weeks and exported to Excel for analysis.

### Metabolic parameter, sympathetic activity, and kidney function study

After 1 week of ANG II infusion and AV-RGS2 transfection, each subject was placed within a NALGENE laboratory metabolic cage (Thermal Scientific) for 24 h and provided standard rat chow and water *ad libitum*. Each cage was given standard rat chow and 250 mL of water. After 24 h, the subject was placed back into a standard rat cage. Water intake was noted. Urine was collected, and 24-h urine volume was measured. Urine was centrifuged and taken to measure sodium, norepinephrine, and creatinine concentration.

Urinary sodium concentration was measured via a flame photometer (Jenway PFP7, Cole-Parmer Ltd., Vernon Hills, IL). Urinary and serum norepinephrine (LSBio, Shirley, MA) and creatinine (Sigma) were measured via ELISA kit. With concentrations of urine sodium and norepinephrine, 24-h urine volume measurements allowed us to measure the 24-h excretion rate of sodium and norepinephrine. 24-h urine volume, urine and serum creatinine concentration, and body weight were used to calculate glomerular filtration rates (GFR) of each subject.

### General procedures during acute volume reflex experiments

#### Tracheostomy

An incision was placed along the midline of the tracheal area, and blunt dissection was performed to reveal the cartilaginous rings of the trachea. A transverse incision on the trachea was made, and a polyethylene tube was inserted and secured. The tube was examined for adequate ventilation by assessing the fogging of the tube and normal respiratory effort, frequency, and depth.

#### Vascular access

An incision was placed along the femoral triangle. The femoral artery and vein pair were exposed and isolated. The femoral artery catheter was assessed for pulsatile flow within the tube, and proper readings were made on the MacLab system monitor (ADInstruments, Colorado Springs, CO). Blood pressure and heart rate measurements were recorded from the arterial catheter. Saline replacement was given through the venous catheter.

### Retroperitoneal access and urostomy placement

While the subject was in a prone position, a series of incisions and blunt dissection along the lumbar region were made to access the retroperitoneal space. The ureter was isolated from surrounding tissues and cut to allow for a PE-10 tube to be inserted. Urine output was assessed by measuring the before-and-after weight of the collecting vial, calculating liquid volume, and dividing by the collection time. Following, kidney weight was also measured to evaluate the volume per time collected per gram of kidney.

### Saline challenge protocol (acute volume reflex)

The subject was given Thiobutabarbital (Inactin hydrate, 2 mL/kg, concentration 0.05 g/mL) via an intraperitoneal injection. Left and right urostomies were provided, and the subject was allowed 15–30 min recovery. Baseline urine output was measured over 15-min intervals with a 1 mL/h normal saline replacement. After baseline measurements, saline replacement was increased to 10% of body weight per hour. Urine output was collected every 5 min until 30 min. Then, saline replacement was reduced to 1 mL/h, and a recovery baseline was collected at two 15-min intervals. Collection tubes were weighed before and after urine collection, allowing us to calculate urine volume from the difference in measurements. Urine flow and sodium concentration changes were noted and calculated throughout the acute experiment.

### Tissue preparation and Western blot

Frozen tissue from the PVN were cut with a cryostat according to a stereotaxic atlas. The brain sections were bilaterally punched using the Palkovits and Brownstein technique (Palkovits, 1983). The punches were homogenized in 100 μL of radioimmunoprecipitation assay (RIPA) buffer containing a 1% protease inhibitor cocktail (Promega, Madison, WI) and phosphatase inhibitor cocktail (ThermoFisher Scientific). Bicinchoninic acid assay (ThermoFisher Scientific) was then completed to measure the protein concentration and calibrate each sample. 4 × loading buffer was added (1:3) to the protein samples and placed within the −80°C freezer for later use.

Western blot protocol included loading protein samples (30–40 μg) onto a sodium dodecyl sulfate polyacrylamide electrophoresis gel and subjecting them to electrophoresis for 120 min at 90 V. The gel was then transferred to a polyvinylidene difluoride membrane for 90 min at 300 milliamps. Initial blocking was completed in Tris-buffered saline with tween (TBST)-milk. Primary mouse antibodies were incubated overnight in the concentrations shown below. Secondary anti-mouse antibody conjugated to Alexa Fluor 680 (1:10000, ThermoFisher Scientific) was incubated over 1.5–2 h and then washed. Fluorescent signals of the blotted membranes were detected using a LICOR scanner (Lincoln, NE) and quantified using ImageJ from NIH. The membranes were analyzed for target protein labeling intensity *versus* a β-actin control.

The following primary antibodies were used: RGS2 (sc-100761, 1:125); G⍺q (sc-136181, 1:125); G⍺s (sc-135914, 1:125); PKC⍺ (sc-8323, 1:125), PKA⍺ (sc-28315, 1:125), and β-actin (sc-398595, 1:1000) (Santa Cruz Biotechnology, Santa Cruz, CA).

### Real-time PCR

Frozen PVN tissue samples were extracted by punching and placed into TRI reagent (Molecular Research Center, Cincinnati, OH) and homogenized for 10–15 s. RNA extractive of each punched tissue was dissolved in DNase/RNase-free water, and reverse transcription was completed. The sample of cDNA was used in quantitative real-time PCR (Applied Biosystems, ThermoFisher Scientific) to have a quantitative measurement for the primers used. The melting curve was analyzed to ensure the quality of the quantitative real-time PCR experiment. RNA primers include RGS2, G⍺q and G⍺s, with β-actin used as a housekeeping primer. Relative expression of target gene was calculated with delta-deltaCt method, which related expression of the target gene to expression of a housekeeping gene (β-actin).

The following primer sequences were used: RGS2-Sense Strand - GCC TGA TGG AGA ACA ACT CTT A; RGS2-Antisense Strand - TCA TCT CAC ACC CTG CTT TC; GNAq-Sense Strand - GCC TGC ATC AGT CAG TAT GT; GNAq-Antisense Strand - GGC TTT CTA GAG CAA GGG ATA G; GNAs-Sense Strand - AGA GGA GAA AGG AGG AGA AGA A; GNAs-Antisense Strand - GCC TCT GTA GCA GGA AGT TAA G; β-actin-Sense Strand - GAG GTA TCC TGA CCC TGA AGT A; β-actin-Antisense Strand - GCT CGA AGT CTA GAG CAA CAT AG.

### Cryostat sectioning and immunohistochemistry

Under deep anesthesia with isoflurane, the subject was perfused through the left cardiac ventricle with heparinized saline followed by 4% paraformaldehyde. The brain was removed, post-fixed with paraformaldehyde, and then placed in 30% sucrose. Brain sections of the PVN (each section 30 µm thick) were cut with a cryostat according to a stereotaxic atlas and preserved in cryoprotectant.

Immunohistochemistry steps included a blocking step (1 h in 10% donkey serum), primary antibody incubation (anti-RGS2, 1:200, MABC1220, Millipore, Sigma) (overnight in 1% donkey serum in PBS-Triton), and secondary antibody incubation (Alexa Fluor 488 donkey anti-mouse secondary antibody, 1:200, 2 h, Jackson ImmunoResearch, West Grove, PA). After washing, the sections were then placed on slides, dried, and covered with a Vectashield mounting medium (Vector Laboratory, Burlingame, CA). Immunofluorescence of RGS2 and mCherry within the PVN (sections from −1.7 mm to −1.8 mm to bregma) were viewed by a Leica fluorescence microscope and captured by a digital camera (Leica, Germany).

### Hypothalamic cell culture

For the cell culture studies, a hypothalamic cell line (mHypoA-POMC/GFP-1, CLU500, CELLutions Biosystems Inc., ON, Canada) was used. We used the mHypoA-POMC cell line as they are a hypothalamic cell line, and they have a common protein expression to other hypothalamic cell lines. However, their unique protein expression (responsible for their neuroendocrine function) should also be considered. We had indiscriminate targeting of the PVN, so all PVN neurons, including POMC neurons, are potentially impacted.

A culture medium of 1% penicillin/streptomycin in Dulbecco’s Modified Eagle Medium (DMEM) was used with fetal bovine serum to grow the cells within the culture flask. Cell cultures were maintained until they reached 60%–70% confluency, and then the cells were plated for the ANG II and AV-RGS2 treatment studies.

Cells were plated for 24–48 h and then starved for another 24 h. Following this, we treated the individual plates with their assigned treatment. Treatments had varying final concentrations of ANG II (6.25, 12.5, 25, 50,100 µM) in DMEM. Each dish contained 5 mL of DMEM. Viral transfection was achieved by adding 50 µL AV-RGS2 (stocking concentration: 10e8, final concentration: 10e6) for −24 h. Cells were scraped from the plates, collected, and used for the mRNA studies. Alongside the ANG II treatments, an additional dish of starved cells was used as a control. This control was treated with 100 µL of only DMEM, while the treated cells had varying concentrations of ANG II in 100 µL DMEM. Gene expression targets were compared to β-actin controls.

### Calcium imaging

For calcium imaging, mHypoA-POMC/GFP-1 cells were pre-incubated with or without AV-RGS2 for 24 h at 37°C in an 8-well Ibidi slide chamber (Ibidi United States, Inc., Fitchburg, Wisconsin). Each well contains 300 µL of DMEM. Viral transfection was achieved by adding 3 µL AV-RGS2 (final concentration: 1e7). After incubation, the cells were loaded with Fluo-3 (5 μM, stocking: 150 μM, add 10 µL into 300 µL medium) (ThermoFisher Scientific) for 30 min at 37°C. At the end of the incubation, cells were washed with DMEM to remove extracellular Fluo-3 and add fresh DMEM (300 µL). Cells were then placed on the stage of a laser confocal microscope (Leica). The confocal calcium image with green fluorescence was taken when the neurons were challenged with ANG II (Final concentration: 1 mM). Fluo-3 was excited by light at 488 nm, and fluorescence was measured at wavelengths of >515 nm using a ×100 objective. Raw data was imported into an Excel file for analysis.

### Statistical analysis

The controls for the *in vivo* experiments included Sprague-Dawley rats without ANG II treatment, and ANG II infusion without AV-RGS2. When comparing to the PVN injection of AV-RGS2, we subjected a control Sprague-Dawley rat to AV-GFP PVN injection. Controls for the *in vitro* experiments include a plate of cells treated only with DMEM medium.

Statistical significance in before-and-after treatments was determined by Student’s paired *t*-test. Student’s t-test was used to calculate differences in samples with two groups. Experiments with 3 groups was analyzed by one-way ANOVA, followed by the appropriate *post hoc* analysis. Two-way ANOVA was used to evaluate the effects of two factors (treatment and time).

Data was assessed prior to parametric analysis if the assumptions of parametric analysis were met. If assumptions were violated, appropriate measures were taken to correct these violations. If the violations remain, nonparametric analysis tools were used instead. Statistical significance was accepted when *p < 0.05*. All data were presented as means ± SE. Data was assessed using Prism 9 (GraphPad Software, La Jolla, CA).

## Results

### General data

Male Sprague-Dawley rats were used and had a mean body weight of −395 g at the time of tissue collection ranging between 14 and 16 weeks of age. Chronic ANG II infusion and AV-RGS2 transfection did not significantly change the body weight among the groups.

### Adenoviral gene transfer of RGS2 within the PVN

**FIGURE 1:**
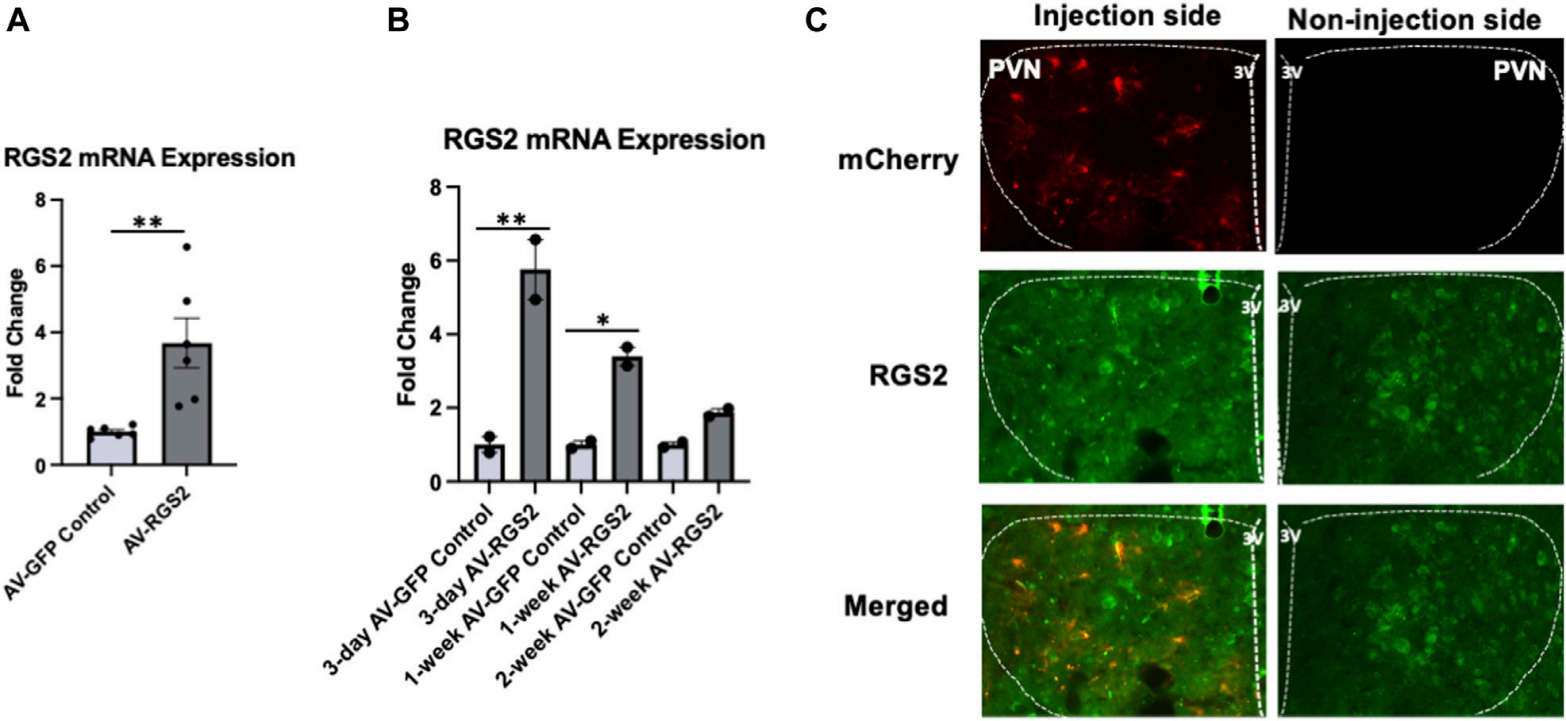
**(A)** The mRNA expression changes of RGS2 within the PVN with AV-GFP or AV-RGS2 transfection, *n* = 6/group. **(B)** The mRNA expression changes of RGS2 within the PVN over time with AV-RGS2 or AV-GFP transfection. RGS2 showed peak expression on the 3^rd^ day, with increased expressions at weeks 1 and 2 of the experiment compared to the AV-GFP injected rats. Four subjects were used for each time point (2 AV-RGS2, 2 AV-GFP). *p < 0.05, **p < 0.01 signifies within groups. **(C)** Histological assessment of AV-RGS2 with mCherry (red) transfection and RGS2 (green) in the PVN area at 6-day post-transfection, X400. *n* = 5. 3V: third ventricle.

We evaluated the efficacy of AV-RGS2 gene transfer in the PVN by comparing RGS2 mRNA levels of the PVN. We completed bilateral adenovirus PVN injections in 12 male Sprague-Dawley rats. 6 rats were transfected with AV-GFP as controls, and the other 6 were transfected with AV-RGS2. Post-transfection tissue collection occurred at 3, 7, and 14 days with two rats in each group for each adenovirus transfection. Analysis of our Sprague-Dawley rat strain showed that the PVN had significantly increased RGS2 mRNA expression in AV-RGS2 transfected rats compared to the AV-GFP transfected rats (Figure 1A). RGS2 showed peak mRNA expression on the 3^rd^ day, with increased expressions at weeks 1 and 2 of the experiment compared to the AV-GFP injected rats. Overall, there was an increase in RGS2 gene expression over the 3 timepoints (Figure 1B).

5 Sprague Dawley rats were unilaterally transfected with AV-RGS2 in the PVN in the left hemisphere, with the right hemisphere being self-control. We compared left *versus* right PVN by the presence and absence of AV-RGS2 coupled with mCherry fluorescent protein. The mCherry and RGS2 fluorescent signal was increased 3-, 6-, 9-, 12-, and 15 days post-transfection. Figure 1C shows the images acquired with mCherry, RGS2, and merged staining within the PVN at 6 days post-transfection.

### Effect of AV-RGS2 on arterial pressure in conscious ANG II infusion rats

**FIGURE 2:**
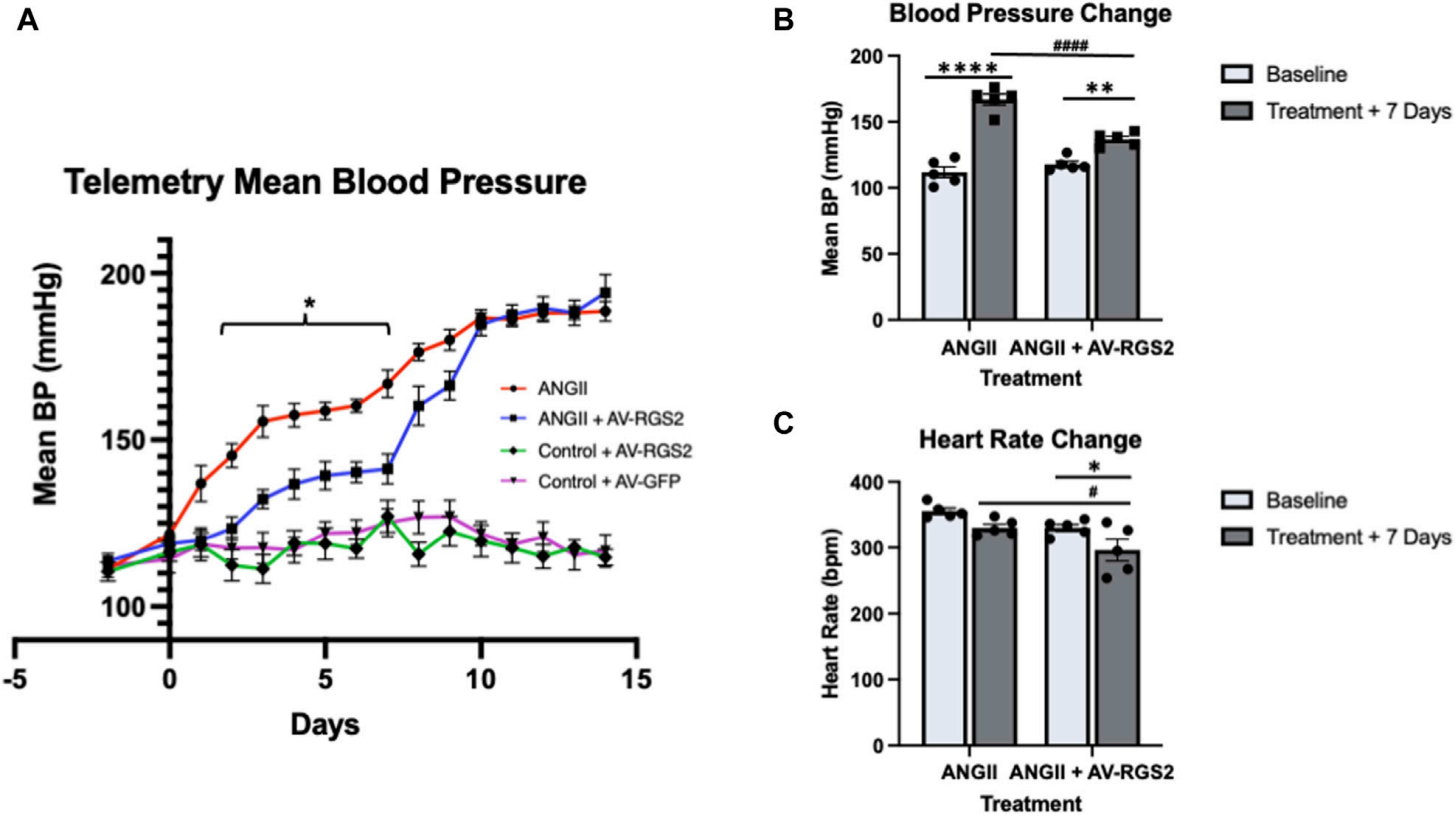
**(A)** The mean blood pressure (BP) changes over a 2-week period. Days 2 through 7 showed a significant reduction of blood pressure in the ANG II + AV-RGS2 groups compared to the ANG II group. *p < 0.05 signifies between groups. **(B, C)**: Depict changes in blood pressure and heart rate at 7-day treatment from baseline. *p < 0.05, **p < 0.01, ****p < 0.0001 signifies within groups, #p < 0.01, ####p < 0.0001 signifies between groups, *n* = 4–5/group.

Baseline blood pressure and heart rate measurements were taken over multiple days after telemetry device insertion recovery and before ANG II infusion (Mean blood pressure: 111.2 +/− 2.3 mmHg). Chronic ANG II infusion significantly increased blood pressure over 2-week (First week: 166.9 +/− 9.3 mmHg; Second week: 188.6 +/− 6.5 mmHg). AV-RGS2 transfection in the PVN significantly attenuated the increase of blood pressure in the ANG II + RGS2 group compared to the ANG II group from day 2 to day 7 (Figure 2A). No differences were noted between when the virus was given, and they showed a similar increase to the hypertensive phenotype occurring early in the 2nd week of the telemetry experiment. The results shown below are in order of the ANG II + AV-RGS2 and ANG II group, respectively (in mmHg). Day 2: 123.4 +/− 7.7 vs. 145.3 +/− 8.0, *p < 0.05*; Day 3: 132.3 +/− 6.4 vs. 155.5 +/− 10.7, *p < 0.05*; Day 4: 136.7 +/− 10.1 vs. 157.4 +/− 8.0, *p < 0.05*; Day 5: 139.3 +/− 9.5 vs. 158.7 +/− 6.0, *p < 0.05*; Day 6: 140.3 +/− 7.0 vs. 160.3 +/− 4.6, *p < 0.05;* Day 7: 141.3 +/− 10.0 vs. 166.9 +/− 9.3, *p < 0.05*. There were no significant differences of blood pressure from day 8 to the end of the experiment. There were no significant differences comparing the normotensive control groups with AV-GFP and AV-RGS2. Day 7 mean blood pressure and heart rate changes from baseline are shown in Figures 2B,C, respectively. There was a significant increase in blood pressure from baseline in both ANG II and ANG II + AV-RGS2 groups. However, the changes in blood pressure in the ANG II + AV-RGS2 group were attenuated compared to those in the group that received ANG II infusion alone. There was a significant decrease in heart rate in the ANG II + AV-RGS2 group *versus* ANG II infusion alone group.

### Effect of AV-RGS2 on the metabolic parameters, sympathetic activity, and kidney function in conscious ANG II infusion rats

**FIGURE 3:**
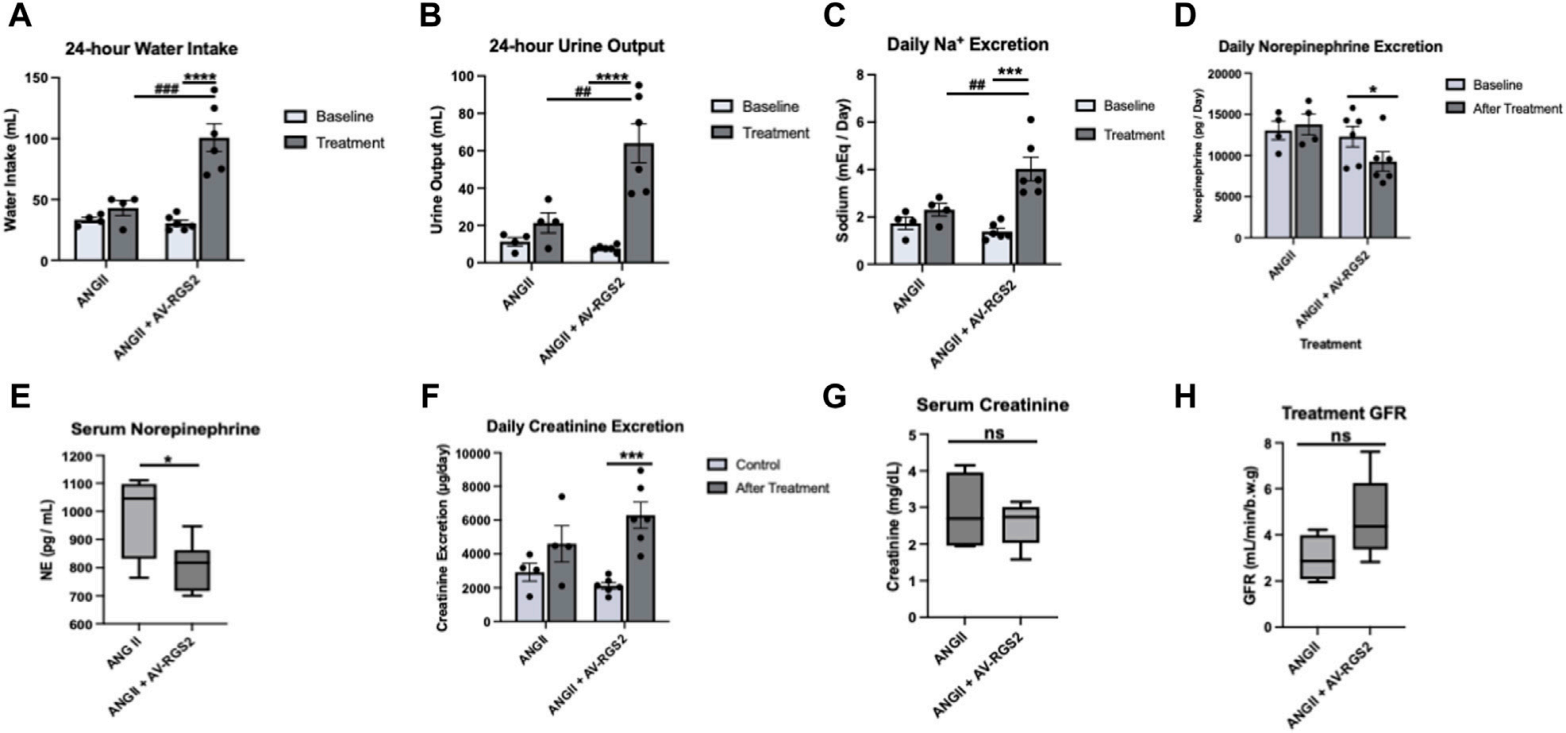
Metabolic changes in ANG II-induced hypertensive rats with or without AV-RGS2 introduced to the PVN. Water intake **(A)**, urine output **(B)**, and urine sodium excretion **(C)** before and after treatment in ANG II and ANG II + AV-RGS2 groups. **(D, E)** showed the changes in serum and urine norepinephrine concentration. **(F–H)** showed the changes in urine excretion and serum concentration of creatinine and GFR. *p < 0.05, ***p < 0.001, ****p < 0.0001 signifies within groups, ##p < 0.01, ###p < 0.001 signifies between groups. n = 4–6/group.

We measured water intake and urine output over 24 h. Water consumption for the ANG II + AV-RGS2 group measured at 100.7 +/− 11.4 mL, which was significantly higher than its pre-treatment baseline (30.5 +/− 2.4 mL, *p < 0.0001*) and the ANG II group (43.0 +/− 6.0 mL, *p < 0.001*) (Figure 3A). Urine output for the ANG II + AV-RGS2 group measured at 64.0 +/− 10.5 mL, which was significantly higher than its pre-treatment baseline (7.6 +/− 0.7 mL, *p < 0.0001*) and the ANG II group (21.4 +/− 5.4 mL, *p = 0.001*) (Figure 3B). Daily sodium excretion significantly increased after treatment in the ANG II + AV-RGS2 group compared to the baseline (4.0 +/− 0.5 vs. 1.4 +/− 0.1 mEq/day, *p < 0.0001*) (Figure 3C). Daily sodium excretion in the ANG II + AV-RGS2 group was significantly increased compared to the ANG II group (4.0 +/− 0.5 vs. 2.0 +/− 0.3 mEq/day, *p < 0.01*).

To evaluate sympathetic activity, assessment of 24-h urine norepinephrine excretion (as sympathetic index) showed that the ANG II + AV-RGS2 group was significantly decreased after treatment compared to the ANG II group (9262.9 +/− 1171.8 vs. 12293.5 +/− 1257.7 pg/day, *p < 0.05*) (Figure 3D). Daily urinary norepinephrine excretion in the ANG II group tended to increase but not reach statistical significance (13021.6 +/− 1119.6 vs. 13783.2 +/− 1267.9 pg/mL, *p > 0.05*). The ANG II + AV-RGS2 group had significantly decreased serum norepinephrine concentration compared to the ANG II group (806.7 +/− 36.0 vs. 991.4 +/− 77.3 pg/mL, *p < 0.05*) (Figure 3E).

To evaluate kidney function, we found that daily urine creatinine excretion was significantly increased in the ANG II + AV-RGS2 group compared to the pre-treatment values (6290.3 +/− 762.1 µg/day vs. 2124.5 +/− 192.8 µg/day, *p < 0.001*) (Figure 3F). Serum creatinine concentration in the ANG II + AV-RGS2 group tended to lower than the ANG II group, but not significantly different between the two groups *(p > 0.05*) (Figure 3G). Calculated glomerular filtration rates tended to be increased in the ANG II + AV-RGS2 group compared to the ANG II group but did not reach significance (*p > 0.05*) (Figure 3H).

### Effect of AV-RGS2 on the acute volume reflex in ANG II infusion rats

**FIGURE 4:**
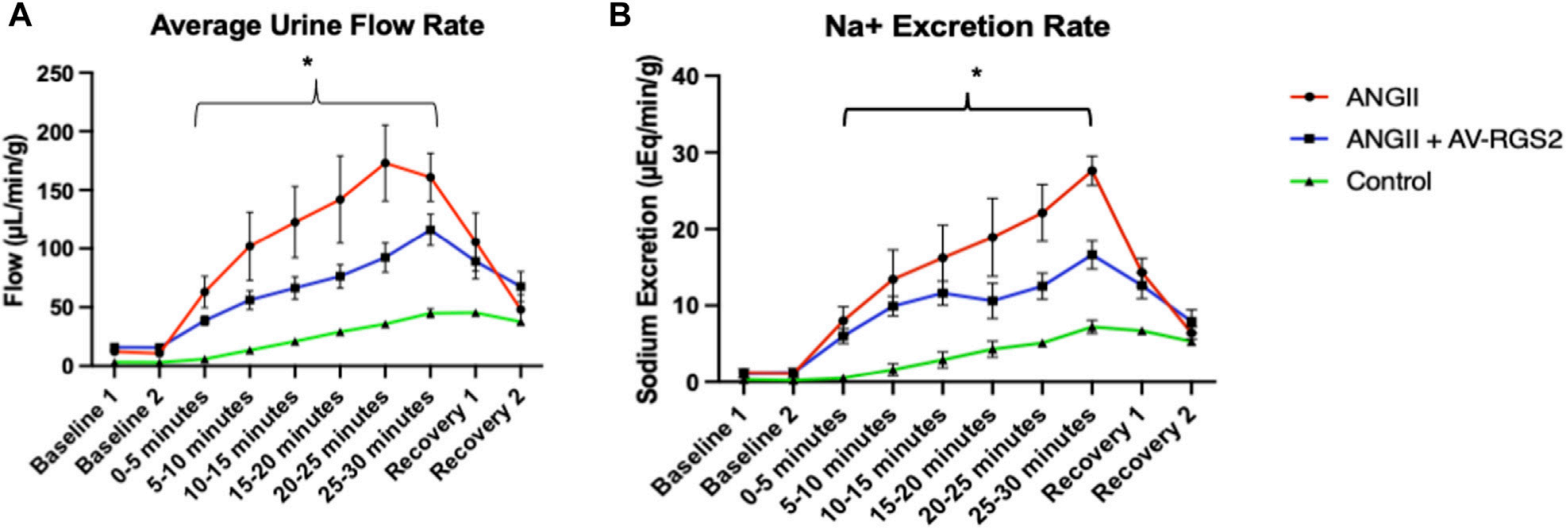
**(A)** The urine flow rate changes when subjected to a 10% body weight per hour saline challenge, measured in uL of urine per minute per gram of kidney. **(B)** The sodium excretion rate with 10% body weight per hour saline challenge, measured in uEq of per minute per gram of kidney. Baseline and Recovery was measured over 15-min intervals. Samples are measured over 5-min intervals. *p < 0.05 compared to the AV-RGS2 and control groups. n = 3–4/group.

Average urine flow and sodium excretion in the acute volume reflex experiment under anesthesia was depicted in Figures 4A,B. There was a significant increased baseline urine volume in the ANG II and ANG II + AV-RGS2 groups compared to the control *(p < 0.05).* The urine flow and sodium excretion responses to saline challenge significantly increased in both ANG II and ANG II + AV-RGS2 groups *(p < 0.05).* However, AV-RGS2 significantly attenuated the urine flow and sodium excretion responses challenged with saline infusion compared to the ANG II infusion group *(p < 0.05).*

### PVN tissue Western blot assessment

**FIGURE 5:**
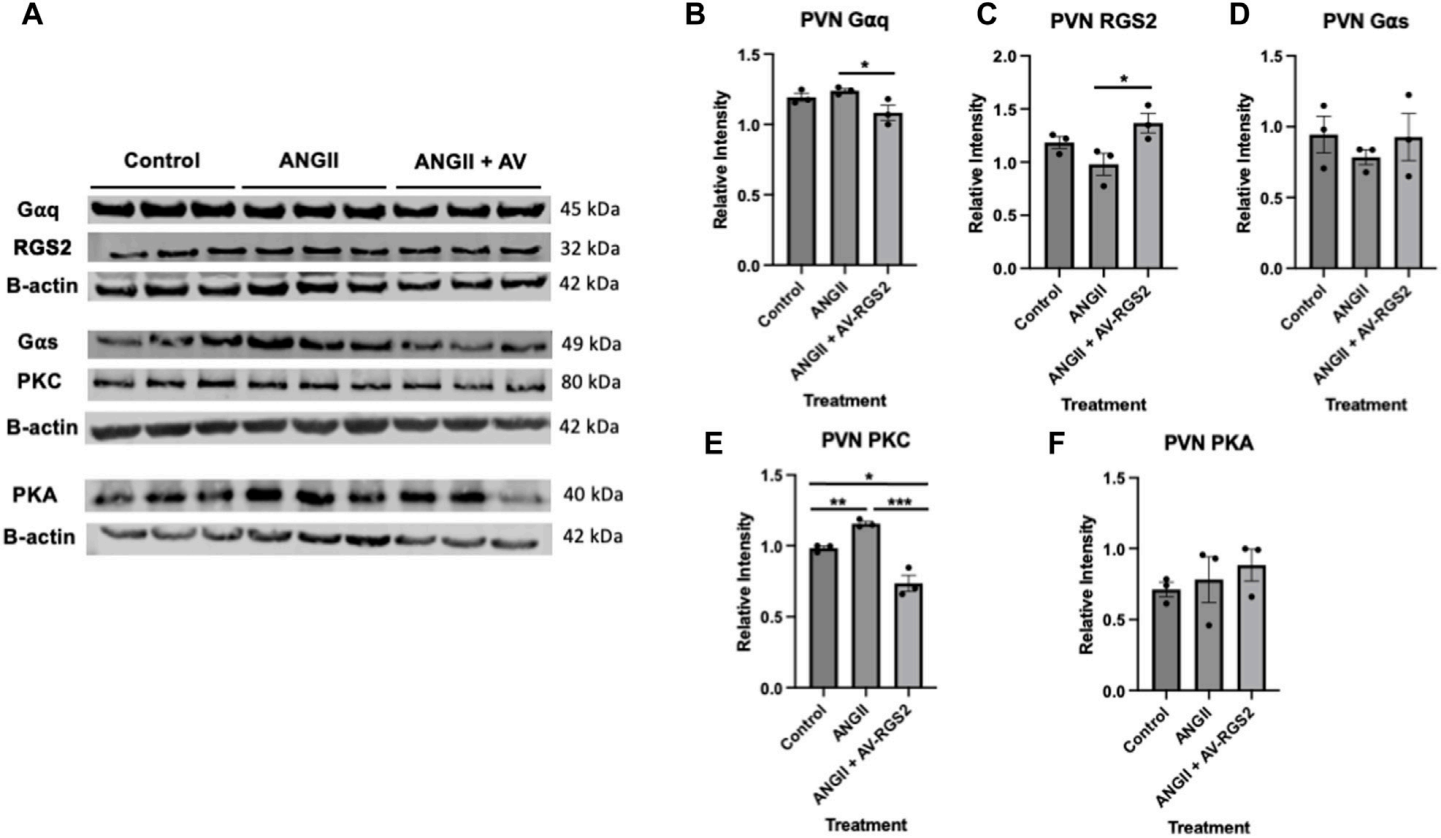
**(A)** The gel images were obtained from the Western blot experiment and used for the data analysis. **(B–F)** Protein expressions of G⍺q, RGS2, G⍺s, PKC and PKA in the PVN with the introduction of AV-RGS2. *p < 0.05, **p < 0.01, ***p < 0.001 signifies between groups. n = 3/group.

Figure 5A shows the gel images obtained from the Western blot experiment and used for the data analysis. Assessment of G-proteins and downstream effectors show that G⍺q, not G⍺s, was significantly decreased in the ANG II + AV-RGS2 compared to the ANG II infusion group (*p < 0.05)* (Figures 5B,D)*.* RGS2 expression in the PVN was shown to be significantly increased in the ANG II + AV-RGS2 compared to the ANG II group (*p < 0.05)* (Figure 5C)*.* We also found that G-protein downstream effector PKC in the ANG II group was significantly increased compared to the control *(p < 0.05).* PKC protein expression were significantly attenuated with AV-RGS2 transfection compared to the ANG II group *(p < 0.05)* (Figure 5E). However, there was no significant change in protein kinase A (PKA) expression in the PVN after AV-RGS2 transfection (*p > 0.05*) (Figure 5F).

### Effect of AV-RGS2 on ANG II-induced G-protein mRNA expression in the hypothalamic cell line

**FIGURE 6:**
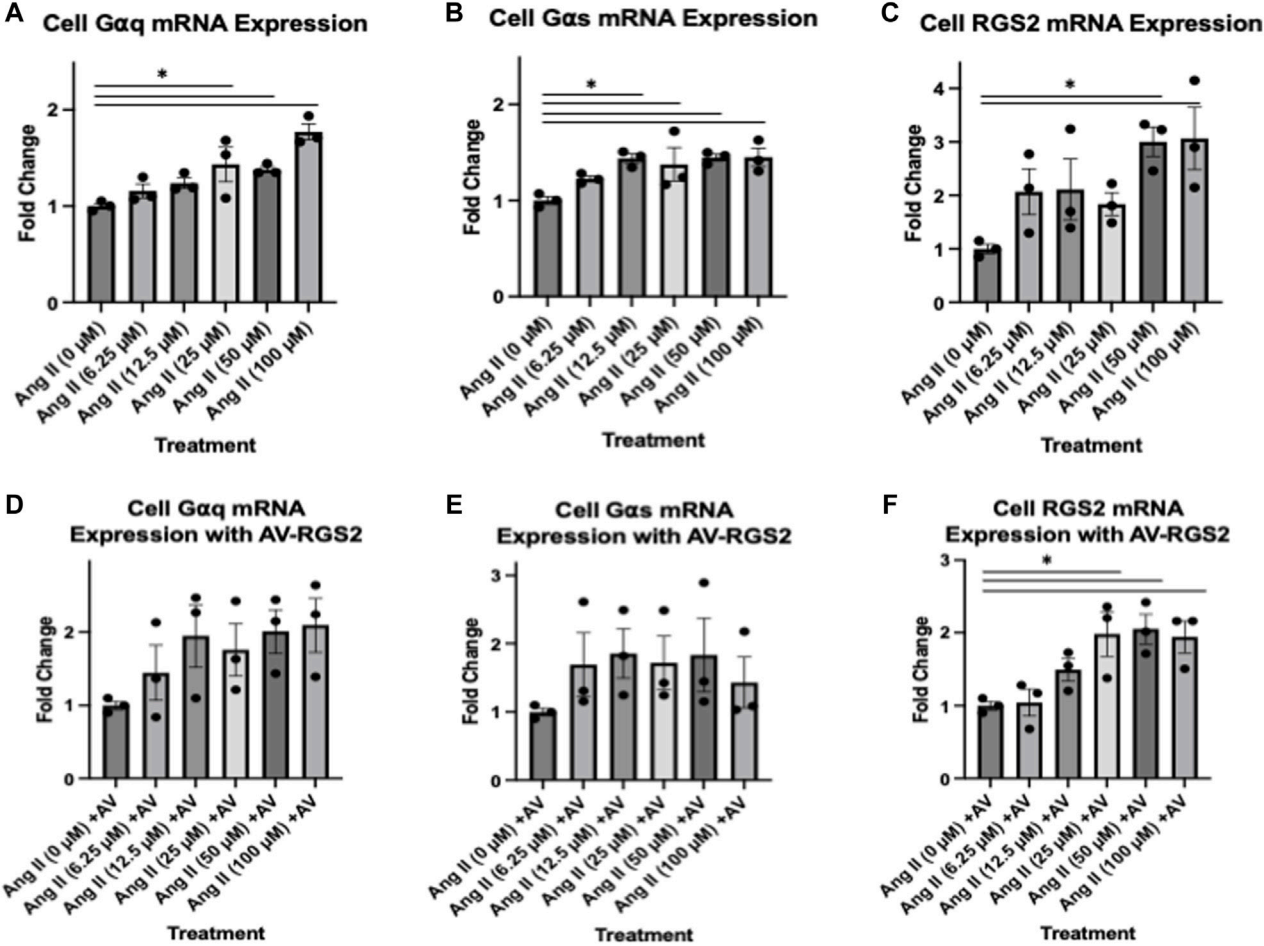
**(A–C)** The changes in mRNA expression in cell culture with treatment of varying concentrations of ANG II. **(D–F)**: Treatments with ANG II with AV-RGS2. There was an increase in mRNA expression as the concentration of ANG II increases. The addition of AV-RGS2 attenuated the fold change seen with ANG II. *p < 0.05 compared to the control without ANG II. n = 3/group.

Real-time PCR analysis showed that treatment of ANG II had a graded increase in G⍺q (Figure 6A), G⍺s (Figure 6B) and RGS2 (Figure 6C) from control, as the concentration of ANG II increased. The collected data had a positive trend and reached statistical significance with ANG II treatment *(p < 0.05)*. In the setting of cell cultures treated with ANG II with AV-RGS2, G⍺q and G⍺s mRNA levels were not significantly different compared to the control *(p > 0.05)* (Figures 6D,E), while RGS2 mRNA showed a similar graded increase (Figure 6F), suggesting AV-RGS2 treatment attenuated the upregulation of G-protein gene expression seen in ANG II treatment.

### Calcium imaging

**FIGURE 7:**
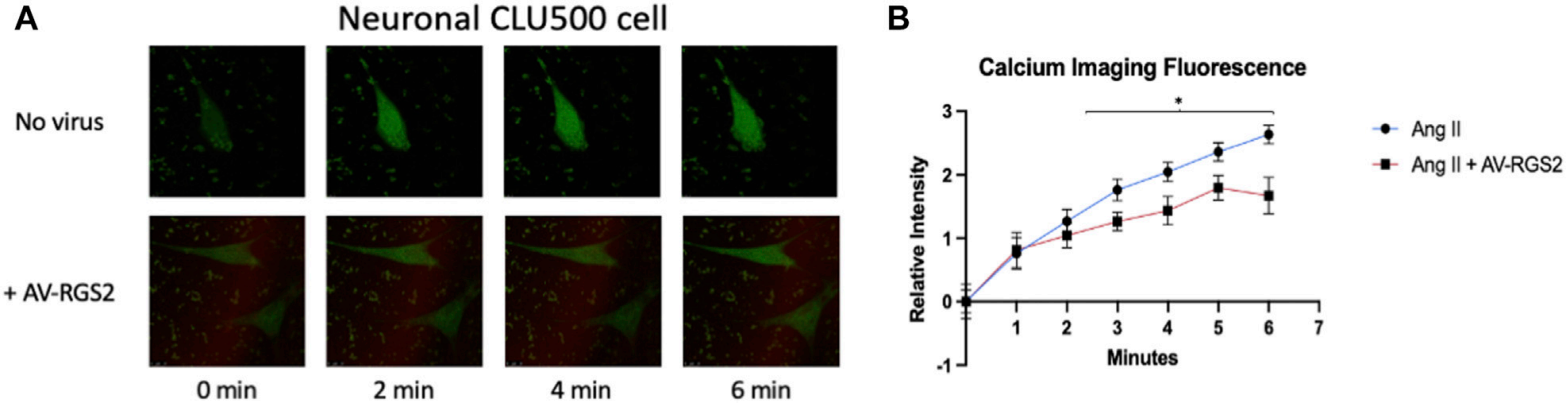
**(A)** The progression of fluorescence over 8 min in cells treated in DMEM with a final concentration of 1 mM ANG II, with/without AV-RGS2 transfection. **(B)** The calculated relative intensity compared to the baseline. Cells treated with AV-RGS2 showed a significant decrease in fluorescence in both duration and intensity. *n* = 3 per group. λ = 488 nm *p < 0.05 compared to the control (ANG II without AV-RGS2).

Treatment of cells with ANG II causes the activation of PKC and influx of calcium into the cytoplasm, which then can be pictured via fluorescence. As RGS2 deactivates the receptor activated by ANG II, we expect to see a reduction of calcium fluorescence in cells treated with ANG II and transfected with AV-RGS2. Confocal imaging was shown in Figure 7A, and fluorescence analysis was displayed in Figure 7B. Analysis showed that AV-RGS2 transfected cells treated with ANG II demonstrated attenuated fluo-3 fluorescence signal compared to the cells only treated with ANG II. Cells treated with AV-RGS2 showed a significant decrease in fluorescence in both duration and intensity.

## Discussion

In this study, AV-RGS2 transfection to the PVN significantly attenuated the increase of mean arterial pressure in ANG II infusion rats from days 2–7 of the 2-week experiment. AV-RGS2 significantly decreased the serum norepinephrine concentration, 24-h urine norepinephrine excretion, and acute volume reflex. AV-RGS2 also increased daily urine volume, water consumption, and sodium excretion in ANG II-infused hypertensive rats. AV-RGS2 transfection within the PVN significantly lower the G⍺q and PKC protein expressions within the PVN in ANG II infusion rats. In hypothalamic cells, AV-RGS2 attenuated ANG II-induced mRNA expression of G⍺q, G⍺s. We also found a attenuated calcium influx in ANG II-treated cells with AV-RGS2 transfection. This data supports the effects of RGS2 in the PVN on blood pressure, sympathetic outflow, and kidney function in hypertensive rats.

Various studies have shown the importance of G-proteins and their impact on cellular signaling (Sriram and Insel, 2018). Of the RGS protein family, RGS2 regulation has been shown to be an emerging target in multiple tissues for various pathologies (McNabb et al., 2020; O'Brien et al., 2019). It is important to investigate the actions of RGS proteins, specifically RGS2, on G⍺q signaling. The activity of RGS2 on G-protein signaling and the cessation of cell signaling may be an important feature in the progression of blood pressure dysregulation. One study shows that a reduced expression of RGS2 within the placenta may contribute to the development of preeclampsia (Perschbacher et al., 2020). RGS2 is also shown to have a role in vascular function, with a reduced expression of RGS2 in the vasculature contributing to hypertension (Osei-Owusu and Blumer, 2015). Additionally, RGS2 maintains a role in renal hemodynamics and contributes to overall renal function (Osei-Owusu et al., 2015).

Other studies have mainly focused on the peripheral mechanisms of RGS2. Still, no studies have shown the central roles of RGS2 and how it impacts blood pressure regulation and kidney function through sympathetic activity modulation. With little to no studies being done on the RGS2 protein on G-protein signaling on the central mechanisms of blood pressure control, we view it as important to examine how these interactions affect sympathetic reflex and the progression of hypertension. Examining the neuronal activity with cell signaling cascade changes in blood pressure management between hypertensive and normotensive subjects may be a critical link needed to help better combat this condition.

We elected to use ANG II infusion to induce a hypertensive state in our rat model. We used ANG II infusion and PVN AV-RGS2 transfection rats in a second group. ANG II infusion quickly induced a hypertensive phenotype in this model. ANG II infusion rats with PVN AV-RGS2 transfection had significantly attenuated the increase of blood pressure compared to the ANG II group. This confirms that overexpression of RGS2 in the PVN attenuates the rise in blood pressure caused by ANG II. The attenuation of the increase of blood pressure in the PVN AV-RGS2 group also suggests that the PVN is a critical central nucleus regulating blood pressure, and RGS2 impacts blood pressure regulation within the PVN.

Our present study shows we can directly alter RGS2 mRNA expression with AV-RGS2 transfection in a rat model, confirmed by mRNA measurement and histological assessment. As expected with adenovirus transfection, there was an acute increase in mRNA expression of RGS2 after 3 days of transfection; then the RGS2 mRNA level gradually decreased over the next 2 weeks. The decreasing level of RGS2 might be because of the immune system clearing the adenovirus transfection. Our hemodynamic study showed that the AV-RGS2 transfection to the PVN significantly reduced mean arterial pressure in ANG II infusion rats in the first week of the 2-week ANG II infusion period. This was consistent with the mRNA study, showing the reduced efficacy of AV-RGS2 transfection after 7 days.

Studies suggest that the dysfunction of sympathetic modulation within the PVN contributes to fluid and electrolyte unbalance and kidney excretory dysfunction, which is seen in hypertension and chronic heart failure (Zheng et al., 2022). Our metabolic study showed urine output and water consumption were increased in the ANG II + AV-RGS2 combined treatment group compared to the ANG II infusion and control groups. This may be caused by an alteration in sympathetic activity from the AV-RGS2 transfection. One characteristic of hypertension is the conservation of sodium. In addition, we saw that daily urine excretion was significantly increased in the ANG II + AV-RGS2 combined treatment model, suggesting sodium retention was relieved by the introduction of AV-RGS2 in the PVN in hypertensive rats.

To examine sympathetic activity changes, 24-h urine norepinephrine excretion was examined. As expected, the excretion rates showed that AV-RGS2 decreased overall norepinephrine excretion. Serum norepinephrine was significantly increased in the ANG II infusion model, which suggested alterations in sympathetic activity. To measure kidney function, urine and serum creatinine concentrations were evaluated and used to determine glomerular filtration. The trends suggest kidney function was improved with the ANG II and AV-RGS2 combined treatment compared to the ANG II infusion model. The data suggests a mild-to-moderate change in kidney function with AV-RGS2 transfection, which was expected as the upregulation of RGS2 impacted overall sympathetic activity and, thus, kidney function in ANG II-induced hypertension.

The PVN is a critical site for the coordination and integration of neurogenic and hormonal actions on the cardiovascular system and the kidney, in response to changes in blood volume (volume reflex) and sodium concentration/osmolality in the body. Several lines of evidence provide credence for the critical role of the parvocellular neurons of the PVN in the neural regulation of fluid balance (Li et al., 2003; Coote, 2005). The specific details of the pathways within the central nervous system and the specific neurotransmitter substances involved in the volume reflex arc have been investigated to some extent. In the experiment to evaluate volume reflex, we saw that the urine flow rate and sodium excretion rates were significantly increased in the ANG II infusion group compared to the control group. ANG II with AV-RGS2 transfection group had attenuated diuresis and natriuresis compared to the ANG II infusion group. These differences suggest that RGS2 alters volume reflex and ANG II-induced fluid responsiveness from the central nervous system.

Furthermore, we examined molecular interactions involving G-protein activation and the activation of downstream effector proteins. ANG II binds to a Gq-protein-coupled receptor, which RGS2 selectively regulates. G-protein activation triggers numerous responses throughout the organism. Gq-protein activates PKC and mobilizes Ca^2+^ by activating phospholipase C, which hydrolyzes PIP3, cleaving it into DAG and IP3 (Mizuno and Itoh, 2009). Gs-protein activates adenylate cyclase activity, which contributes to cAMP production, activating PKA. Gi-protein inhibits this pathway by inhibiting adenylate cyclase (Sassone-Corsi, 2012). This signaling cascade is an important factor of heightened cellular activity, causing several proteins to be transcribed relating to cell cycle progression, proliferation, growth factors, metabolism, neurotrophy, and cell survival (Wang et al., 2018). With G-proteins being present within all cell types, it is plain to see their vital role in cell signaling. Understanding brain G-protein subunits, specifically Gαq/Gαs, is important in how they are directed to alter sympathetic activation and cardiovascular parameters. With this, we elected to evaluate the changes in G-protein and downstream signaling in the PVN as we manipulated ANG II and RGS2.

Our PVN tissue protein analysis showed decreased changes in G⍺q but not G⍺s with AV-RGS2 transfection, further aiding our statement regarding RGS2 selectivity for Gq-protein interaction. As a downstream effector protein of Gq-protein activation, PKC significantly increased with ANG II infusion and decreased with AV-RGS2 transfection compared to the ANG II infusion group. This is expected as RGS2 returns to active G⍺q to its inactive state, halting the downstream actions directly involving PKC. In addition, we evaluated changes in PKA, which is downstream of Gs-protein activation. No changes were present in PKA protein expression in the PVN.

To further examine the role of RGS2 on the ANG II-G-protein signaling in our mouse hypothalamic cell line, we found increased G-protein mRNA expression as ANG II treatment concentration increased. AV-RGS2 treatment reduced the G-protein mRNA expression induced by ANG II treatment. These *in vitro* studies further support that RGS2 interacts with G-proteins and returns them to an inactive state, halting the downstream signaling cascade expected with ANG II-G-protein activation. Further, ANG II acts on a signaling pathway that is expected to increase intracellular calcium, so we should expect an increase in calcium flux by adding ANG II. RGS2 should decrease the duration and intensity of calcium flux by limiting the action of ANG II by Gq-protein deactivation. We used confocal microscopy to measure calcium flux in cells treated with or without AV-RGS2 in response to ANG II. We have demonstrated that AV-RGS-treated cells had a significantly reduced fluorescence compared to those without AV-RGS2 treatment. This suggests an attenuation in calcium flux in the AV-RGS2 treated group, which is the expected result when Gq-protein activation time is decreased with increases in RGS2 protein.

## Perspectives

Our results describe a novel role of RGS2 in the PVN for regulating blood pressure, sympathetic activation, and kidney function in hypertension. These findings show that the action of RGS2 on ANG II-induced Gq-protein activation modulates blood pressure, sympathetic activation, and kidney function in hypertension. Our study provides new insights into the central regulation of blood pressure and potential targets to treat hypertension.

## Limitations and alternative approaches

Many factors influence the overall blood pressure control of an organism. In this study, we are investigating only few impacting factors of blood pressure regulation. In addition, among the receptors and proteins investigated, many isoforms and subtypes of these receptors have differing ligand-receptor interactions and downstream signaling. While between organisms, the tissue-specific receptors are generally conserved, there are subtle differences that may impact overall results. Further investigation is needed to better understand how acting of specific receptors or proteins can have off-target effects. The jump between rodent and human models of hypertension may contribute to a variation of results. Some models of hypertension do not accurately display a phenotype that can be characterized as essential hypertension, which may impact results, especially when comparing essential hypertension in humans.

Additional limitations include our use of an adenovirus as a vector, which has a higher immunogenic response in comparison to adeno-associated virus, which may contribute to unwanted physiological responses. We also used only male models, and thus, sex as a biological variable must be noted. Additional female subjects would have to be used to exclude sex as a biological variable in addition to ANG II infusion and AV-RGS2 transfection. In addition, we used daily sodium excretion from urine to assess sodium handling, while true sodium balance must take account for sodium intake. However, no considerable changes in food intake were noted. Finally, we used some controls for our telemetry study, yet we did not have an ANG II infusion with AV-GFP control to determine vector impacts on ANGII infusion. These limitations will be considered in future studies.

## Data Availability

The original contributions presented in the study are included in the article/Supplementary Material, further inquiries can be directed to the corresponding author.
