# Supplementary material for: Effects of regulator of G protein signaling 2 (RGS2) overexpression in the paraventricular nucleus on blood pressure in rats with angiotensin II-induced hypertension
Source: Front Physiol. 2024 Jun 21;15:1401768. doi: 10.3389/fphys.2024.1401768 (PMC11224644; doi:10.3389/fphys.2024.1401768)
Supplement: Supplementary file 1 [file Presentation1.PPTX]

## Slide 1
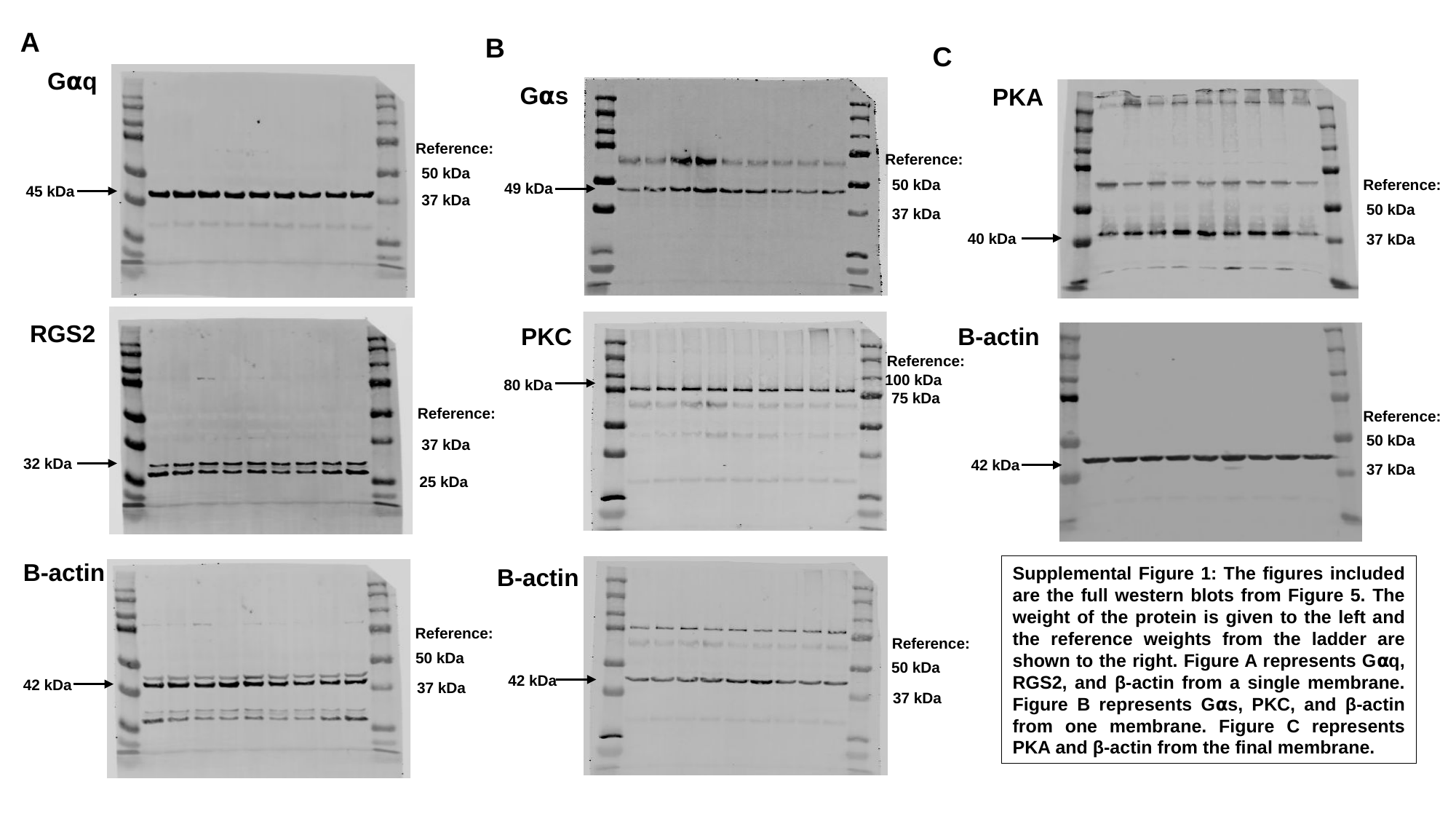

A
B
C
G⍺q
G⍺s
PKA
Reference:
Reference:
50 kDa
50 kDa
Reference:
49 kDa
45 kDa
37 kDa
50 kDa
37 kDa
40 kDa
37 kDa
RGS2
PKC
Β-actin
Reference:
100 kDa
80 kDa
75 kDa
Reference:
Reference:
50 kDa
37 kDa
32 kDa
42 kDa
37 kDa
25 kDa
Β-actin
Supplemental Figure 1: The figures included are the full western blots from Figure 5. The weight of the protein is given to the left and the reference weights from the ladder are shown to the right. Figure A represents G⍺q, RGS2, and β-actin from a single membrane. Figure B represents G⍺s, PKC, and β-actin from one membrane. Figure C represents PKA and β-actin from the final membrane.
Β-actin
Reference:
Reference:
50 kDa
50 kDa
42 kDa
42 kDa
37 kDa
37 kDa
